# Retraction Note: Ibrutinib facilitates the sensitivity of colorectal cancer cells to ferroptosis through BTK/NRF2 pathway

**DOI:** 10.1038/s41419-024-06498-9

**Published:** 2024-02-07

**Authors:** Jin-Feng Zhu, Yi Liu, Wen-Ting Li, Ming-Hui Li, Chao-Hui Zhen, Pei-Wei Sun, Ji-Xin Chen, Wen-Hao Wu, Wei Zeng

**Affiliations:** 1 Department of General Surgery, Shenzhen University General Hospital, 518055 Shenzhen, Guangdong Province P.R. China; 2 Department of Gastrointestinal Surgery, Shunde Hospital, Southern Medical University (The First People’s Hospital of Shunde, Foshan), Foshan, Guangdong Province P.R. China; 3 Department of Cardiothoracic Surgery, Shenzhen University General Hospital, 518055 Shenzhen, Guangdong Province P.R. China; 4 Department of Pathology, Shenzhen University General Hospital, 518055 Shenzhen, Guangdong Province P.R. China; 5 Department of Ultrasound, Shenzhen University General Hospital, 518055 Shenzhen, Guangdong Province P.R. China; 6 Department of Oncology, Shunde Hospital, Southern Medical University (The First People’s Hospital of Shunde, Foshan), 1 Jiazi Road, 528000 Foshan, Guangdong Province P.R. China

**Keywords:** Diseases, Medical research

Retraction to: *Cell Death and Disease* 10.1038/s41419-023-05664-9, published online 23 February 2023

The Editors-in-Chief retracted this article because of concerns regarding a number of figures presented in this work. These concerns call into question the article’s overall scientific soundness and its authorship. An investigation conducted after its publication discovered similarities between images in this work and images in [1],

The panel labelled RSL3 in Figure 6E and the panel labelled HONE-1 (Lung) in Figure 6G in [1];

The panel labelled Erastin in Figure 6E and the panel labelled SUNE-1 (Lung) in Figure 3E in [1];

The Editors-in-Chief therefore no longer have confidence in the integrity of the research presented in this article. The authors did not respond to the correspondence from the publisher about this retraction.

## References

[1] Li C-W, Zheng J, Deng G-Q, Zhang Y-G, Du Y, Jiang H-Y (2022). Exosomal miR-106a-5p accelerates the progression of nasopharyngeal carcinoma through FBXW7-mediated TRIM24 degradation. Cancer Sci.

